# Higher choroidal thickness and lower choriocapillaris blood flow signal density based on optical coherence tomography angiography in diabetics

**DOI:** 10.1038/s41598-021-85065-0

**Published:** 2021-03-11

**Authors:** Yaoli Zhang, Yuanjun Qin, Shuaishuai Wang, Yuyan Liu, Xinyu Li, Xufang Sun

**Affiliations:** 1 Department of Ophthalmology, Tongji Hospital, Tongji Medical College, Huazhong University of Science and Technology, 1095 Jie-fang Road, Wuhan, Hubei China; 2 Department of Ophthalmology, Sir Run Run Shaw Hospital, Zhejiang University School of Medicine, Hangzhou, Zhejiang China; 3 University of Massachusetts Amherst, Amherst, MA USA

**Keywords:** Retinal diseases, Eye diseases

## Abstract

Diabetes mellitus (DM) is one of the fastest growing chronic diseases in the world and one of the main causes of vision loss. Whether or not diabetic choroidopathy (DC) is involved in the initiation and progression of diabetic ocular complications needs to be explored. We included 54 diabetic eyes from 36 diabetic patients, and 54 healthy eyes from 32 control subjects after propensity scores matching. All of the subjects were given pupil light and dark adaptation examination and optical coherence tomography angiography (OCTA). Scotopic pupil diameter (SPD), pupil contraction amplitude, and velocity of pupil contraction of the diabetic group were significantly lower than that of the healthy control group (P < 0.05).Choroidal thickness at temporal quadrant (at 750 μm) and superior quadrant (at 1500 μm and 2250 μm) increased in diabetic group compared to control group(P < 0.05).In the diabetic group, choriocapillaris blood flow signal density (CCBFSD) in the macular area (diameter = 2000 μm) were significantly decreased compared with the healthy control group (P < 0.05). Apparent changes in pupil and choroidal blood flow were observed in the diabetic patients.

## Introduction

Diabetes mellitus (DM) is one of the fastest growing chronic diseases in the world and one of the leading causes of vision loss^1^. According to the World Health Organization (WHO), the total number of people with DM will grow from 171 million in 2000 to 366 million in 2030^2^. At present, the pathological mechanisms of diabetic retinopathy are mainly explained by the effects of vascular leakage, vascular abnormalities, neuronal cell dysfunction, and inflammatory mechanisms^3^.

The concept of diabetic choroidopathy was proposed by Saracco et al. as early as 1982^4^. Histopathological studies revealed a variety of choroidal changes secondary to DM^5–7^. The choroid accounts for more than 85% of the retinal blood supply and supplies all the nutrients for the retinal pigment epithelium and photoreceptors^8,9^. It has been reported that photoreceptors die rapidly when the choroidal blood flow decline drastically^10^. The regulation of choroidal blood flow includes neuromodulation, autoregulation, and local choroidal ganglion regulation^11–13^.

Diabetic autonomic neuropathy (DAN) refers to a disorder of the autonomic nerves caused by DM, which involves diseases related to multiple systems including orthostatic hypotension, tachycardia, gastroparesis, diarrhea, constipation, etc. Its ocular complications include a reduction of scotopic pupil diameter and an Argyll-Robertson-like pupil^14^. Studies have shown that nerve modulation of the uvea affects blood flow in the iris, ciliary body, and choroid^15,16^.

Both the choroid and the iris are parts of the uvea, and they have an inseparable relationship in anatomy, structure, and development of each other. Although there is a lot of evidence confirming the presence of pupillary lesions and changes in choroidal thickness caused by diabetic autonomic nerve damage^17–21^, little research on diabetic choroidal blood flow and its relation to pupil lesions is currently available. Previous studies have shown that the blood flow of choroidal capillaries in diabetic patients is significantly lower than that of healthy people^22,23^. Some studies have shown that their blood flow further decreases as the severity of the disease increases^24^.

In recent years, advances in optical coherence tomography (OCT) technology has revolutionized retinal research and clinical practice^25^. OCT angiography (OCTA) is a recently developed technique that allows noninvasive visualization of vascular structures^26–28^. Moreover, the higher speed of the new OCT equipment has facilitated the optical reconstruction of complex vascular structures^25^. This feature of OCTA makes it an extremely valuable tool for studying choroidal blood flow^29^.

## Results

### Patient demographics and clinical characteristics

A total of 108 eyes (no DR, 31 eyes; mild NPDR, 19 eyes; moderate NPDR, 4 eyes; control group, 54 eyes) were included for final analysis after PSM. The demographic and clinical characteristic details can be identified in Table 1.

**Table 1:** Demographic and clinical characteristics of patients by group.

	Before PSM			After PSM		
	Healthy group (eyes = 89)	Diabetic group (eyes = 84)	P	Healthy group (eyes = 54)	Diabetic group (eyes = 54)	P
Age (years)	40.94 ± 10.99	50.54 ± 8.94	< 0.01	44.70 ± 11.50	48.33 ± 9.35	> 0.05
Female/Male	49/40	29/55	< 0.01	26/28	23/31	> 0.05
BCVA (logMAR)	− 0.02 ± 0.05	0.00 ± 0.08	< 0.05	− 0.03 ± 0.06	0.01 ± 0.09	< 0.05
IOP (mmHg)	15.15 ± 2.49	16.49 ± 2.63	< 0.01	15.12 ± 2.55	15.64 ± 2.55	> 0.05
SE (D)	− 1.18 ± 1.70	− 0.32 ± 1.48	< 0.01	− 0.61 ± 1.52	− 0.51 ± 1.53	> 0.05
AL	23.59 ± 1.18	23.37 ± 1.04	> 0.05	23.42 ± 1.11	23.36 ± 1.09	> 0.05
Stage (NDR/mild NPDR/moderate NPDR)	–	44/32/8	–	–	31/19/4	–

*BCVA* best corrected visual acuity, *IOP* intraocular pressure, *SE* spherical equivalent, *AL* axial length.

### Pupillometry

The details of the diabetic and healthy control groups for pupil diameters at light intensities of 0 cd/m^2^, 1 cd/m^2^, 10 cd/m^2^, and 100 cd/m^2^, pupil contraction amplitude (PCA), velocity of pupil contraction (PCV) and velocity of pupil dilation (PDV) are shown in Table 2. This study showed that the scotopic pupil diameter (SPD), PCA and PCV of the diabetic group were significantly lower than that of the healthy control group (4.96 ± 0.78 vs 5.31 ± 0.89, 1.36 ± 0.32 vs 1.53 ± 0.31, 4.84 ± 0.68 vs 5.17 ± 0.90, respectively; P < 0.05). The pupil size at light intensities of 1 cd/m^2^, 10 cd/m^2^, 100 cd/m^2^, and PDV were not significantly different from those in the healthy control group (P > 0.05).

**Table 2:** Results of pupillometry for the patients by group after PSM.

	Control group (n = 54)	Diabetic group (n = 54)	P value
SPD (mm)	5.31 ± 0.89	4.96 ± 0.78	< 0.05
PD1 (mm)	3.82 ± 0.90	3.50 ± 0.76	> 0.05
PD2 (mm)	2.90 ± 0.48	2.83 ± 0.49	> 0.05
PD3 (mm)	2.52 ± 0.32	2.53 ± 0.38	> 0.05
PCA (mm)	1.53 ± 0.31	1.36 ± 0.32	< 0.05
PCV (mm/s)	5.17 ± 0.90	4.84 ± 0.68	< 0.05
PDV (mm/s)	2.01 ± 0.32	2.12 ± 0.54	> 0.05

*SPD* scotopic pupil diameter, *PD1* pupil diameter at light intensity of 1 cd/m^2^, *PD2* pupil diameter at light intensity of 10 cd/ m^2^, *PD3* pupil diameter at light intensity of 100 cd/m^2^.

#### Optical coherence tomography

**Figure 1:**
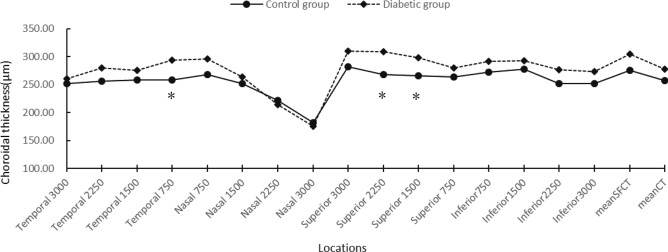
CT (μm) in 17 locations by group after PSM. *Significantly related on the 0.05 level (both sides).

Choroidal thickness (CT) at different locations of the fundus of the diabetic and the control groups is shown in Fig. 1. Results showed that CT at temporal quadrant (at 750 μm) and superior quadrant (at 1500 μm and 2250 μm) increased in diabetic group compared to control group (P < 0.05). There were no statistically significant differences in mean subfoveal choroidal thickness (SFCT), mean choroidal thickness, and CT in other locations and between two groups (P > 0.05).

#### Choriocapillaris blood flow signal density (CCBFSD)

**Figure 2:**
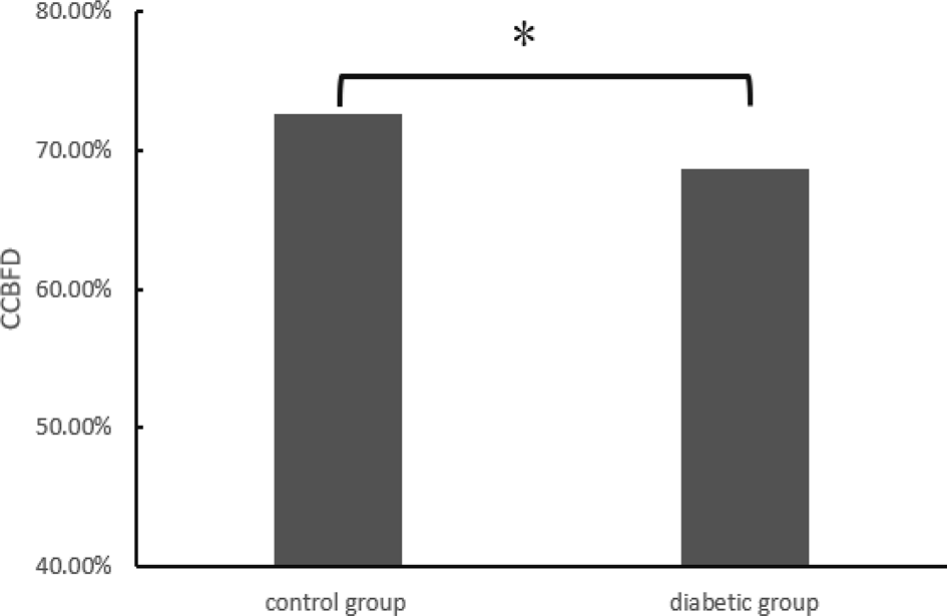
Choriocapillaris blood flow signal density by group after PSM. *Significantly related on the 0.05 level (both sides).

CCBFSD in the macular area in the diabetic group were significantly lower than that in the healthy control group (P < 0.05). The details are shown in Fig. 2.

## Discussion

Our study showed that SPD, PCA, and PCV in the diabetic group were all lower than those in the healthy control group, which is consistent with results of previous studies^20,21^. We consider that this difference may result from pupillary autonomic nerve disorders in diabetic patients, suggesting that hyperglycemia damages the autonomic nerves of the iris.

We found that the CT at temporal quadrant (at 750 μm) and superior quadrant (at 1500 μm and 2250 μm) in the diabetic group significantly increased compared with the healthy control group. For this point, previous studies have had different results. Some clinical findings suggest choroidal thinning of early diabetic retinopathy (DR)^17,18,30,31^; however, some conflicting results suggest early thinning, late thickening^19^, or completely contrast choroidal thickening^32–34^. Some of these studies have their own characteristics that may explain the heterogeneity of the results. Querques et al. only compared CT in the central region^18^, and we know that choroidal changes in diabetes not only affect the central region but, more importantly, affect the circumference. The Vujosevic’s study included all stages of DR, including some patients with PDR^17^. In Esmaeelpour’s study, some patients had a history of hypertension and were taking medications^30^, which could affect choroidal circulation.

OCTA, as a method for non-invasive visualization technique of fundus blood vessels, can provide a more comprehensive assessment of choroidal circulation. Compared with OCT, it provides a richer amount of blood flow information. Although OCTA has been frequently used clinically, it is rarely used as a tool for the study of DC. This is an innovative aspect of this research.

Compared with previous studies, choriocapillaris blood flow signal density assessment method has been further updated. We use the self-programmed code to position and analyze each image. First of all, we perform batch analysis of pictures based on code, which can reduce manual errors and have good repeatability. Secondly, the code can accurately calculate the blood flow signal of each picture, thus forming 512 B-scan pictures into a "map" representing the choriocapillaris blood flow volume in the macular area, and finally calculating the percentage of choriocapillaris capillary blood signal density in this area.

Our study showed a lower CC perfusion in diabetic eyes compared with healthy eyes. Previous study has illustrated that eyes with NPDR are affected by macular hypoperfusion and photoreceptor damage and a lower CC perfusion may be associated with a greater damage of photoreceptor^35^. We speculate that there are two reasons for decreased CCBFSD in the macular area with diabetes. Firstly, decreased CCBFSD in diabetic patients is caused by vascular damage and blood flow decreasing due to long-term hyperglycemia. Secondly, although it is not realistic to completely exclude the effect of bleeding and exudation on signal acquisition, the patients we included were all patients with no DR, and mild or moderate DR whose bleeding and exudation were not severe. It should be noted that there is no evidence that slight retinal hemorrhage could significantly affect the collection of choroidal blood flow signals.

At the same time, the increase of choroidal thickness and the decrease of SPD occurred in the diabetic group, and both were regulated by autonomic nerves, suggesting that this may be related to diabetic autonomic nerve damage. As we know, the decline of SPD is related to autonomic nerve damage. Furthermore, the middle vascular layer and large vascular layer of choroid also have autonomic and trigeminal innervation. The mechanism and their correlation deserve further research.

A previous report by Borrelli et al. demonstrated that a further dilation of the area occupied by medium-sized and larger-sized choroidal vessels and/or choroidal stroma appears to be associated with a progressive reduction in CC perfusion in healthy eyes^36^. It might suggest that an increased choroidal thickness may be associated with a lower CC perfusion, which is not surprising. Increased choroidal thickness and decreased choriocapillaris perfusion in patients with central serous chorioretinopathy, similar to the results in our study of patients with diabetes^37,38^. This reminds us that there may be some similar mechanisms in these two diseases. Firstly, thicker choroid may be caused by increased choroidal vessel wall permeability in an inflammatory state in both diseases. Secondly, choriocapillaris hypoperfusion may be caused by mechanical stress resulting from compression based on focal or diffusely enlarged choroidal vessels^37^.

Compared with previous studies, we better matched certain factors, such as age, gender, intraocular pressure, axial length and refractive status, to minimize the effect of these confounding factors on CT. At the same time, the OCT examination is performed at a constant time period of a day, which reduces the influence of the daily rhythm on the choroidal measurement.

At the same time, we also need to consider the following points. First, differences in choroid assessment methods may lead to conflicting results. Diabetic choroidal vascular abnormalities are most common in the mid-circumference. However, OCT examinations focus on the macular/foveal area, so the results of these studies may not accurately reflect the actual situation. Second, OCTA has limitations as well: that is, only the velocity of blood flow within a certain range can be detected by the equipment. The velocity of choroidal capillaries may occasionally be too low to generate a disassociation signal, preventing OCTA from recording blood flow signals in capillaries. Thirdly, compared with the choroidal capillary layer, it is difficult to accurately evaluate blood flow of medium and large choroidal vessels because of the occlusion of retinal vessels and choroidal capillaries, and high blood flow velocity in these vessels.

In short, with the advent of the latest OCTA technology, DC has become one of the most compelling diseases to study for ophthalmological researchers. Nonetheless, the relationship between DC and DR, and the role of DC in diabetic ocular diseases remain unknown. This important field requires further research.

## Materials and methods

### Subject groups

This study was performed according to the Declaration of Helsinki and was approved by the Ethics Committee of Tongji Hospital. Informed consent was obtained from all study participants before examination.

#### Subjects

We included 47 diabetic patients (84 eyes) and 48 healthy people (89 eyes) from the Department of Ophthalmology and Department of Endocrinology of Tongji Hospital, Tongji Medical College of Huazhong University of Science and Technology. Because the baselines of age, intraocular pressure, sex and spherical equivalent (SE) in two groups were not well matched, propensity scores matching was used. 54 diabetic eyes from 36 diabetic patients and 54 healthy eyes from 32 control subjects were included for final analysis after propensity scores matching, which were used to balance the two groups according to several baseline variables, including age, sex, intraocular pressure, SE. DM was diagnosed according to the 1999 WHO diagnostic criteria.

#### Inclusion criteria

Diabetic patients were included who had no signs of DR, or those diagnosed with mild or moderate non-proliferative diabetic retinopathy (NPDR).

#### Exclusion criteria

These included: (1) hypertension, kidney disorders, and other systemic diseases that may affect fundus circulation; (2) glaucoma, cataract, and history of ocular surgery; (3) vitreous opacity; (4) high myopia; (5) age-related macular degeneration, choroidal neovascularization, central serous retinal choroidopathy, and other eye diseases that affect the fundus circulation; (6) DM patients treated with laser and intraocular anti-vascular endothelial growth factor (VEGF), or macular edema; (7) patients with a history of ocular trauma.

### Study procedure

All subjects underwent visual acuity (VA), intraocular pressure (IOP), slit lamp examination, ophthalmoscopy, optometry, fundus photography, examination of pupil light and dark adaptation, parafoveal 3 × 3 mm^2^ OCTA scanning, and foveal OCT line scan. Subjects with B-scan OCT image quality lower than 30 and OCTA quality lower than 35 were excluded.

Pupil function tests were performed using a comprehensive pupillometer (Vision Monlter Mon2013K, Metrovision, France). Examinations were performed by the same ophthalmologist, including pupil diameter (PD) at different light intensities (0 cd/m^2^, 1 cd/m^2^, 10 cd/m^2^, and 100 cd/m^2^), pupil contraction amplitude (PCA), velocity of pupil contraction (PCV), velocity of pupil dilation (PDV).

The 3 × 3 mm^2^ parafoveal area with 512 B-scan OCT images per eye scanned by Heidelberg SPECTRALIS OCT device (Heidelberg Engineering GmbH, Heidelberg, Germany) was used to analyze the choriocapillaris blood flow signal in this region. The OCT B-scan was performed horizontally and vertically through the fovea. CT at the nasal, superior, temporal, and inferior choroid quadrants (at 750 μm, 1500 μm, 2250 μm and 3000 μm intervals from the center of the fovea) and subfoveal choroidal thickness (SFCT) were recorded. Mean CT is their average. Choroidal thickness was measured as the perpendicular distance between the hyperreflective outer border of the retinal pigment epithelial/Bruch’s membrane layer and the sclero-choroidal interface, manually drawn blindly by the examiner. OCTA and OCT examinations were performed using the Heidelberg SPECTRALIS OCT equipment in 8:30 a.m.–13:00 p.m. for reducing choroidal changes with daily rhythm.

**Figure 3:**
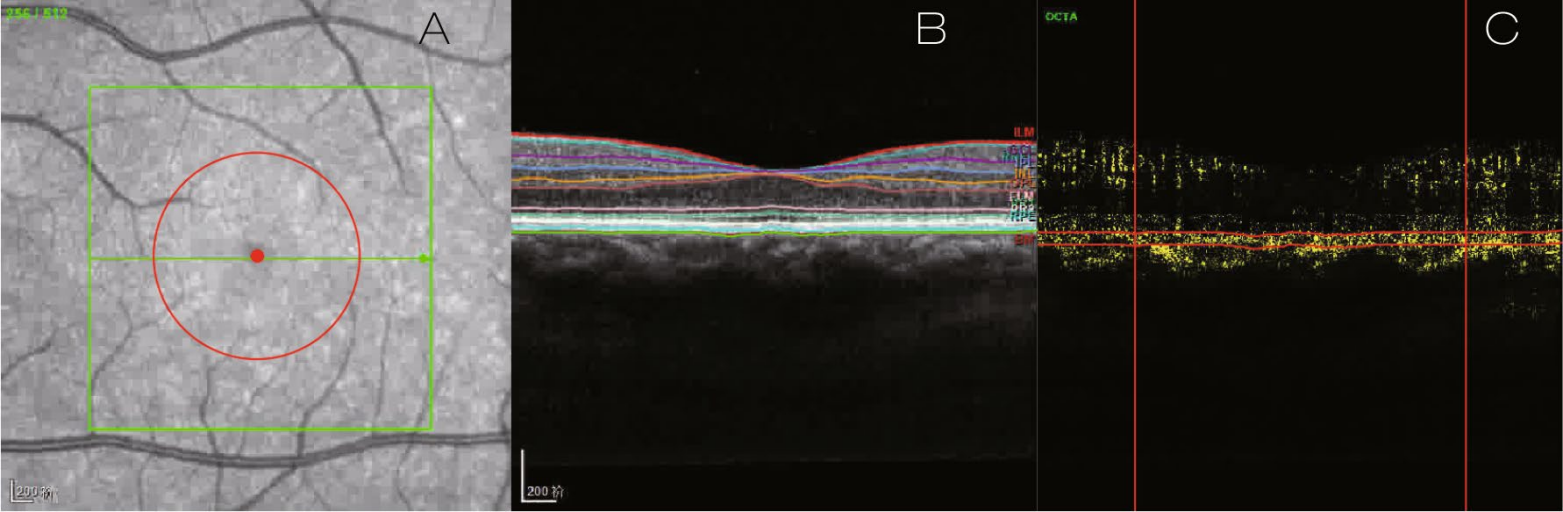
The 256th OCTA image. Yellow signals indicate blood flow signals. (**A**) En face image of structural OCT. Green rectangle is the scanned 3 × 3 mm^2^ area. The red circle area indicates the 2000-μm-diameter macular area where we calculate CCBFSD. (**B**) OCT B-scan segmented image with yellow blood flow signal after manual adjustments. (**C**) OCTA B-scan image with yellow blood flow signal after self-written code process. The two horizontal red lines are inner and outer boundaries of choriocapillaris layer respectively. These two vertical red lines indicate the boundary of the 2000-μm-diameter macular area we calculated in the B scan OCTA image. The area enclosed by the above four lines is the area and blood flow signal we need to calculate in the 256th OCTA image.

We used SPECTRALIS Software Version 6.9a to layer the acquired images. We would make manual adjustments if the automatic segmentation was incorrect, and then used the self-written code to calculate choriocapillaris blood flow signal density (CCBFSD) in the macular area (centered by the fovea, 2000 microns in diameter), which is the percentage of the yellow signal to the total area. Choriocapillaris was segmented with an inner boundary Bruch membrane and an outer boundary 49 μm below the Bruch membrane. The positioning method of the choroidal capillary area for calculation in the 256th image of 512 pictures is described detailly in Fig. 3. We took out the calculation area picture enclosed by the four red lines in Fig. 3C, converted it into a grayscale image, and then binarized it (cut off value is 30), and calculated the total pixels of the image and the pixels occupied by the blood flow signal. We performed the above operations on all 512 OCTA images in one eye, and finally obtain: CCBFSD = total blood flow signal pixels of 512 images /total pixels of 512 images. The image positioning and calculation operations were all completed in batches by self-edited code.

### Statistical analysis

All the data in this study were analyzed using IBM SPSS Statistics ver.26.0 (IBM, Armonk, NY, USA). The propensity score was estimated using a logistic regression model with 1:1 nearest neighbour matching without replacement, based on an acceptable caliper width of 0.25 times. Kolmogorov–Smirnov test was used for the normality test. Independent sample t-test was used to compare the data between these two groups that satisfied the normal distribution; if it did not, non-parametric test was used. Chi-square test was used for gender comparison between the two groups. Measurement data were expressed as mean ± standard deviation. P < 0.05 was considered statistically significant.

## Data Availability

The datasets used and/or analysed during the current study are available from the corresponding author on reasonable request.
